# Biogenic silver nanoparticles based on *trichoderma harzianum*: synthesis, characterization, toxicity evaluation and biological activity

**DOI:** 10.1038/srep44421

**Published:** 2017-03-16

**Authors:** Mariana Guilger, Tatiane Pasquoto-Stigliani, Natália Bilesky-Jose, Renato Grillo, P. C. Abhilash, Leonardo Fernandes Fraceto, Renata de Lima

**Affiliations:** 1 Federal University of São Carlos, Sorocaba campus, Rodovia João Leme dos Santos, km 110, 18052-780, Sorocaba, Brazil; 2 Laboratory of Biotechnology, University of Sorocaba, Rodovia Raposo Tavares, km 92, 18023-000, Sorocaba, São Paulo, Brazil; 3 Center of Natural and Human Sciences, Federal University of ABC, Santo André, Brazil; 4 Institute of Environment & Sustainable Development, Banaras Hindu University, Varanasi 221005, India; 5 Laboratory of Environmental Nanotechnology, Department of Environmental Engineering, São Paulo State University (UNESP), Sorocaba, São Paulo, Brazil

## Abstract

White mold is an agricultural disease caused by the fungus *Sclerotinia sclerotiorum*, which affects important crops. There are different ways of controlling this organism, but none provides inhibition of its resistance structures (sclerotia). Nanotechnology offers promising applications in agricultural area. Here, silver nanoparticles were biogenically synthesized using the fungus *Trichoderma harzianum* and characterized. Cytotoxicity and genotoxicity were evaluated, and the nanoparticles were initially tested against white mold sclerotia. Their effects on soybean were also investigated with no effects observed. The nanoparticles showed potential against *S. sclerotiorum*, inhibiting sclerotia germination and mycelial growth. Nanoparticle characterization data indicated spherical morphology, satisfactory polydispersity and size distribution. Cytotoxicity and genotoxicity assays showed that the nanoparticles caused both the effects, although, the most toxic concentrations were above those applied for white mold control. Given the potential of the nanoparticles against *S. sclerotiorum*, we conclude that this study presents a first step for a new alternative in white mold control.

The challenges facing agriculture in the 21^st^ century include climate change, restrictions on agricultural expansion, and resistant pests and diseases that damage crops. It is predicted that the global population will increase by a third between 2009 and 2050, with an associated demand for 3 billion tons of cereals^1^. Soybean is one of the world’s most economically important crops, and Brazil is the second largest producer and the largest exporter^2^. The crop grows rapidly under different environmental conditions and is superior to all other crops as a protein source. It is considered an important component of the global food supply^3^. However, in recent years soybean crops have been affected by a pathogenic fungus, *Sclerotinia sclerotiorum*, which causes the disease known as white mold. This lowers yields and contaminates the soil due to the ability of the organism to produce resistant sclerotia that can remain viable in the soil for as long as eleven years, leading to potential epidemics^4^. Although different types of chemical and biological control can be employed against this disease, none has been shown to be able to inhibit germination of the sclerotia^5^,^6^.

Nanotechnology, a recent introduction to agricultural science, appears to offer a number of promising solutions^7^. These include the modified release of pesticides and fertilizers^8^,^9^,^10^,^11^,^12^, nanosensors for the detection of pesticides in the environment^13^, and the control of phytopathogens^14^,^15^,^16^. Among the metallic nanomaterials, silver nanoparticles (AgNPs) are highlighted due to their antimicrobial properties^17^. Different techniques can be used to synthesize AgNPs, the most widely used being chemical methods, due to the ease of preparation^18^. However, these procedures use large amounts of toxic chemicals. As an alternative, biogenic synthesis of AgNPs offers a way of reducing the use of chemicals, since it employs reducing agents and stabilizers extracted from organisms including bacteria, fungi, and plants^19^.

Fungi are of particular interest as reducing agents and stabilizers in the biogenic synthesis of nanoparticles, due to rapid mycelial growth, production of large quantities of protein, easy handling of the biomass, and economic viability^20^. *Trichoderma harzianum*, a filamentous mycoparasite employed as a biological control agent, is an example of a fungus used as a reducing agent and stabilizer in the biogenic synthesis of AgNPs^21^,^22^.

The application of silver nanoparticles to control phytopathogens has been shown to be viable, as reported by Mishra *et al*.^16^, who synthesized biogenic AgNPs using an agriculturally important bacterium as a reducing agent. The nanoparticles showed promising results for controlling the phytopathogenic fungus *Bipolaris sorokiniana*, which affects wheat crops. Kim *et al*.^14^, reported the antifungal potential of silver nanoparticles for the *in vitro* control of several species of phytopathogenic fungi, with the inhibitory effects varying according to the pathogen and the concentration and type of nanoparticles.

The aim of the present work was to synthesize biogenic silver nanoparticles using a filtrate of the biological control fungus *Trichoderma harzianum* as a reducing agent and stabilizer, for a potential use against the phytopathogenic fungus *Sclerotinia sclerotiorum*, responsible for white mold disease. There are no previous reports of the application of biogenic silver nanoparticles synthesized using *Trichoderma harzianum* for controlling the germination of the sclerotia of *Sclerotinia sclerotiorum*. Therefore, it is indispensable to investigate the toxicity of this new nanomaterial ensuring the knowledge of its future safety issues.

The biogenic silver nanoparticles (AgNP-T) prepared were characterized in order to confirm the synthesis and determine parameters including the size distribution, polydispersity index, zeta potential, concentration, and morphology. Cytotoxicity and genotoxicity assays were performed using animal cells, plant cells, and microorganisms. These included *Allium cepa* chromosome aberration assays, evaluation of cell viability using the methylthiazol tetrazolium (MTT) reduction test, evaluation of apoptosis and oxidative stress using imaging cytometry (Tali^TM^), comet assays using cell cultures, and determination of minimum inhibitory concentrations in *Escherichia coli, Staphylococcus aureus*, and *Candida albicans* cultures. Given the importance of understanding the possible effects of the nanoparticles on soil microbiota, molecular evaluations of soil microbiota were performed, using real-time PCR to quantify the DNA of nitrogen cycle bacteria exposed to different concentrations of nanoparticles. Finally, initial tests were performed to evaluate the potential of the nanoparticles to control the mycelial growth and sclerotia germination of *Sclerotinia sclerotiorum*, as well as the effects on the germination and growth of soybean seedlings, with the aim of obtaining a new and effective system for the control of agricultural pests.

## Results and Discussion

### Physico-chemical characterization of the biogenic silver nanoparticles

**Figure 1:**
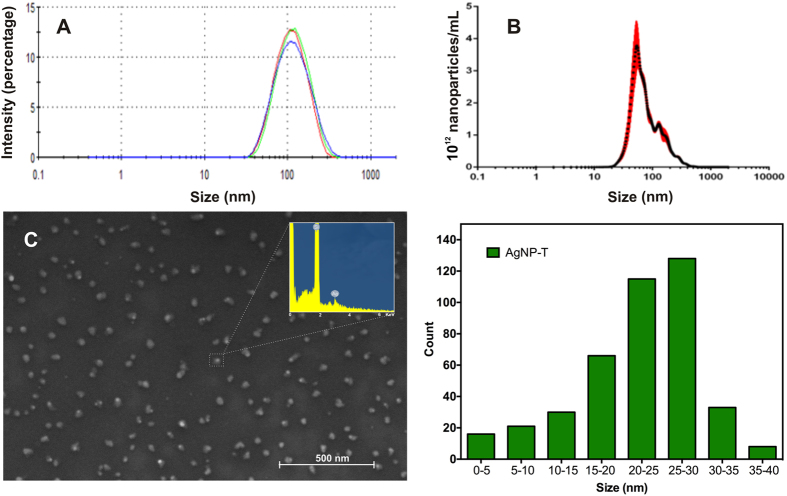
Size distribution of silver nanoparticles biogenically synthesized with the fungus *Trichoderma harzianum*. (**A**) dynamic light scattering (DLS, n = 3), (**B**) nanoparticle tracking analysis (n = 3), and (**C**) scanning electron microscopy (SEM) coupled to energy dispersive X-ray spectroscopy (EDS) analysis.

The size distribution of the biogenic silver nanoparticles (AgNP-T) was determined using dynamic light scattering (DLS), nanoparticle tracking analysis (NTA), and scanning electron microscopy (SEM). The average diameter determined by DLS was 100.7 ± 0.3 nm and the distribution profile was monomodal (Fig. 1A). Using NTA, the average size distribution was 58.0 ± 4.0 nm (Fig. 1B), with a concentration of 3.16 × 10^12^ nanoparticles/mL (NPs/mL). SEM image revealed spherical nanoparticles, without any aggregates, and a size distribution between 20 and 30 nm (Fig. 1C). These differences in the size distributions were due to the characteristics of the techniques and the procedures used to prepare the samples, since the samples were dried prior to the SEM analyses and maintained in dispersion for the other methods. The nanoparticles showed a zeta potential of −6.85 ± 1.45 mV by microelectrophoresis technique, and a polydispersity index of 0.27 ± 0.03 by DLS, indicating low size variability and good physico-chemical stability^23^. It has been reported previously that the use of biological stabilizers during synthesis can improve the stability and dispersion of nanoparticles^24^,^25^. In addition, energy dispersive X-ray spectroscopy (EDS) analysis coupled to SEM showed a peak characteristic of silver, confirming the presence of the metal in the nanoparticles (Fig. 1C).

### *Allium cepa* chromosome aberration assays

**Figure 2:**
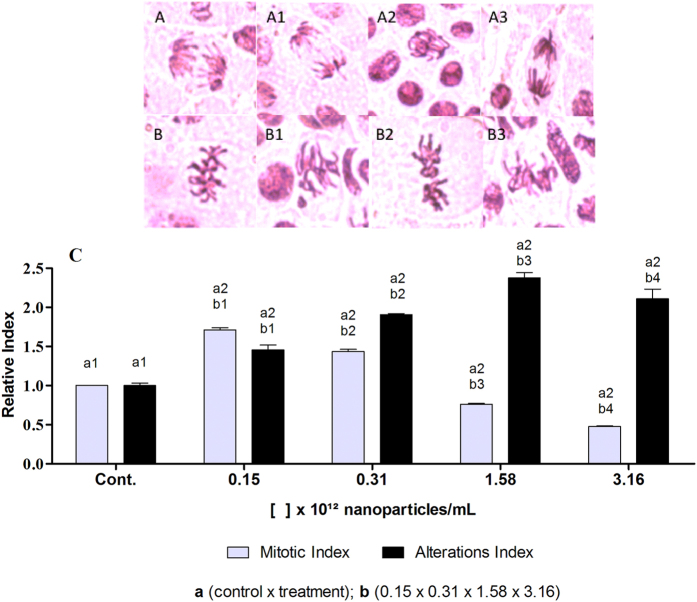
*Allium cepa* chromosome aberration analysis of biogenic silver nanoparticles (AgNP-T) at different concentrations, with 24 h of exposure. (**A**) Normal anaphase at 40x magnification (A1, A2, and A3 alterations); (**B**) Normal metaphase at 40x magnification (B1, B2, and B3 alterations); (**C**) mitotic index and DNA alteration index. Different letters indicate different comparisons and different numbers indicate significant differences (p < 0.05).

The *Allium cepa* tests were performed using different concentrations of nanoparticles (0.15 × 10^12^, 0.31 × 10^12^, 1.58 × 10^12^, and 3.16 × 10^12^ NPs/mL). The biogenic silver nanoparticles induced changes in the mitotic index and the alteration index, compared to the negative control (exposure to ultrapure water). In the case of the mitotic index, use of the nanoparticles at the two lower concentrations (0.15 × 10^12^ and 0.31 × 10^12^ NPs/mL) resulted in significantly higher values than obtained for the negative control, while significantly lower values were obtained at the two higher concentrations (1.58 × 10^12^ and 3.16 × 10^12^ NPs/mL) (Fig. 2). This was probably due to different phenomena and interactions occurring at the different nanoparticle concentrations. In the case of the alteration index, use of the nanoparticles resulted in significantly higher values than the negative control, at all the concentrations studied (Fig. 2).

Panda *et al*.^26^, used the *Allium cepa* test to evaluate the genotoxic potential of biogenic silver nanoparticles synthesized using the male inflorescence of the plant *Pandanus odorifer*. Use of the nanoparticles resulted in chromosome alteration indices that were significantly higher than obtained with the negative control, but lower than those obtained with commercial nanoparticles (Sigma-Aldrich, size <100 nm, purity 95.5%). Lima *et al*.^27^, used the *Allium cepa* test with biogenic silver nanoparticles synthesized from the fungus *Fusarium oxysporum*, which induced significantly higher alteration rates, compared to the negative control.

Several studies have reported differences in mitotic and alteration indices according to nanoparticle concentration and the duration of exposure^28^,^29^,^30^. However, it is important to note that biogenic silver nanoparticles can vary in terms of their characteristics, with different levels of toxicity due to different synthesis conditions and the use of reducing agents and biological stabilizers that result in different coatings^31^.

### Cell viability assays using the methylthiazol tetrazolium (MTT) reduction test

**Figure 3:**
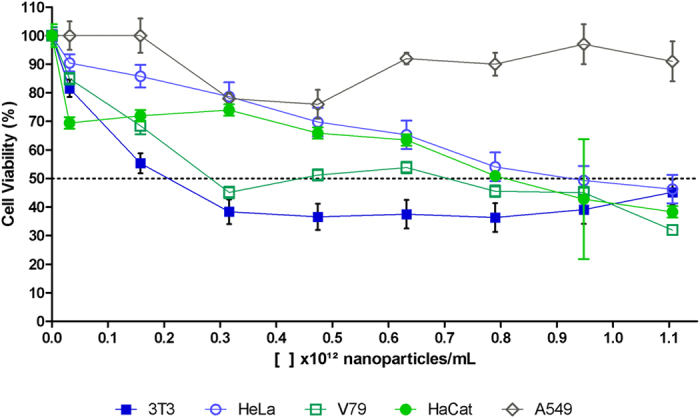
Analysis of cell viability using the MTT reduction test applied to 3T3, HeLa, HaCaT, V79, and A549 cells exposed for 24 h at 37 °C to biogenic silver nanoparticles (AgNP-T) in 96-well plates at a concentration of 2 × 10^4^ cells/well. The dashed line represents the concentration of nanoparticles necessary to achieve 50% cell inhibition (IC_50_).

The MTT test was used to determine the cytotoxic potential of the biogenic silver nanoparticles. The results showed that the nanoparticles were cytotoxic towards the 3T3, HeLa, HaCat, and V79 cell lines (Fig. 3), with the IC_50_ values varying according to the cell line. The 3T3 cells showed the greatest sensitivity to the nanoparticles, with the lowest IC_50_ of 0.21 × 10^12^ NPs/mL, while the HeLa and A549 cells showed greater resistance, with IC_50_ of 0.91 × 10^12^ NPs/mL for the HeLa cells, while this parameter was not reached for the A549 cells at the concentrations tested (Fig. 3). According to Mukherjee *et al*.^32^, different cell lines show different responses when exposed to silver nanoparticles, with some being more sensitive and others having greater resistance. In this study, the effects of exposure were investigated using five different cell lines, employing the same culture medium, so the observed differences were not due to the dispersion medium, but rather to the nature of the cells.

Braroo *et al*.^28^, investigated the cytotoxicity towards human keratinocytes (HaCaT) of biogenic silver nanoparticles synthesized using leaves from the plant *Ocimum sanctum*. Three different concentrations were tested, and an IC_50_ value was found for the highest exposure concentration. Han *et al*.^33^, observed cytotoxicity of biogenic silver nanoparticles synthesized using the bacterium *Escherichia coli*, as well as nanoparticles produced by chemical synthesis, towards epithelial cells of human pulmonary adenocarcinoma (A549). The results of the MTT assay indicated that the toxicity was concentration-dependent for both types of AgNPs, and that the biogenic AgNPs were significantly more toxic at lower concentrations, compared to the AgNPs synthesized chemically.

It should be noted that in some cases there might be doubts concerning use of the MTT reduction assay, because nanoparticles can interfere in the absorption of light at the MTT wavelength, leading to overestimation of viability. These interferences vary according to the type and size of the nanoparticles^34^,^35^,^36^,^37^. Hence, in order to ensure that the observed results were significant, the viability of the cells was also evaluated by imaging cytometry to confirm cell death and determine whether it was caused by apoptosis or cell necrosis.

### Evaluation of apoptosis and oxidative stress by imaging cytometry (Tali^TM^)

The analysis of cell viability and death, considering apoptosis or necrosis, was assessed using imaging cytometry, exposing the cells to a concentration of 0.93 × 10^12^ NPs/mL, which was found to cause substantial cell death in the evaluation using the MTT test. The exposure time was 24 h, as in the MTT assays.

**Figure 4:**
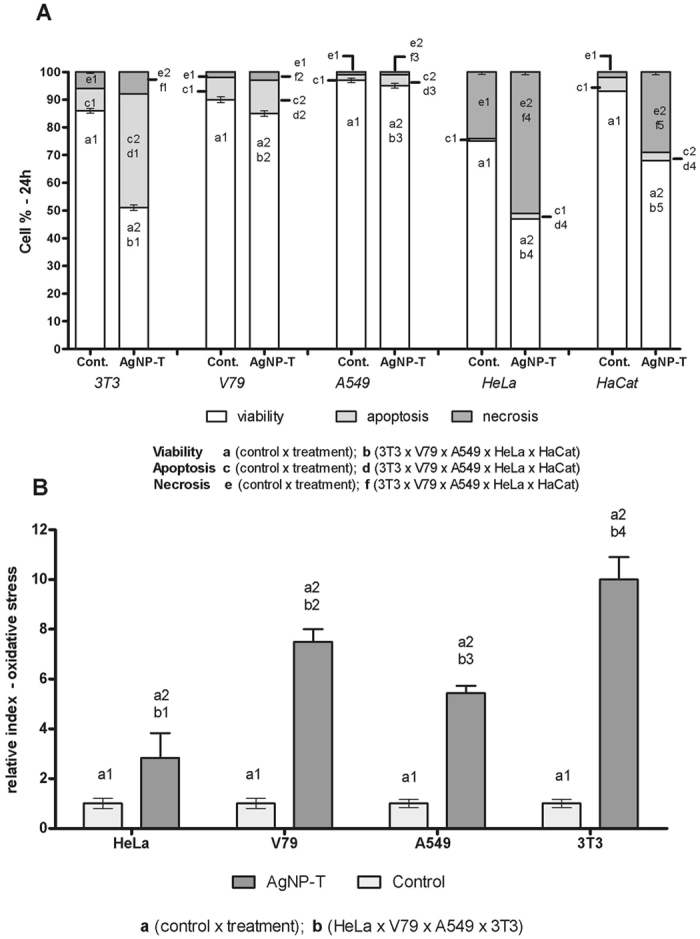
Imaging cytometry analysis. (**A**) Cell viability, apoptosis and necrosis of the 3T3, V79, A549, HeLa, and HaCaT cell lines exposed to 0.93 × 10^12^ NPs/mL of biogenic silver nanoparticles (AgNP-T) for 24 h at 37 °C. (**B**) Relative oxidative stress index for the 3T3, V79, A549 and HeLa cells exposed to AgNP-T at a concentration of 0.93 × 10^12^ NPs/mL for 1 h at 37 °C. Different letters indicate different comparisons and different number indicate significant statistical differences (p < 0.05).

Necrosis and apoptosis are morphologically distinct types of cell death that are caused by different stimuli in the forms of external or internal factors. Necrosis is usually due to the loss of membrane integrity, while apoptosis is caused by chromatin condensation following DNA damage. According to Kumar *et al*.^37^, cells exposed to silver nanoparticles can exhibit both necrosis and apoptosis, depending on nanoparticle size, concentration, and exposure time. Here, the results obtained for AgNP-T using imaging cytometry showed different responses according to the cell lines used, in agreement with the MTT test. Comparison of the results of the two tests showed that the cytometry analysis (Fig. 4A) indicated a lower cell death rate (at the concentration of 0.93 × 10^12^ NPs/mL tested), compared to MTT test (Fig. 3), except for HaCaT cells.

The A549 cells showed greater cell viability, with no significant differences between the control cells and those exposed to AgNP-T, although there was a small increase in apoptosis in cells exposed to AgNP-T. The substantial increase in apoptosis observed for the 3T3 cells reflected the low IC_50_ value found in the MTT analysis.

The HaCaT and HeLa cell lines showed greater responses to exposure to the nanoparticles at the concentration tested, with marked necrosis (Fig. 4A), especially in the case of the HeLa cells (tumoral uterine epithelium). Other work has found increased necrosis when the cells were exposed to silver nanoparticles for long periods^37^.

Mizushima *et al*.^38^, reported a high rate of apoptosis of 3T3 cells, with the internalization of AgNPs increasing autophagosomes, autolysosomes, and oxidative stress in the cells. In this study, it was therefore decided to evaluate oxidative stress as the cellular response to contact with the nanoparticles.

Studies have shown that AgNPs lead to the production of reactive oxygen species and consequently to cytotoxicity due to apoptosis following the destabilization of cellular homeostasis, especially in the case of nanoparticles smaller than 100 nm^37^,^39^,^40^,^41^,^42^,^43^,^44^,^45^. Hence, the cells were placed in contact with AgNP-T at a concentration of 0.93 × 10^12^ NPs/mL for 1 h, after which different responses were observed according to the cell line (Fig. 4B). Comparison of the response to oxidative stress with the results for apoptosis and necrosis showed that the cells exposed to greater oxidative stress had higher rates of apoptosis (3T3, V79, and A549 cells). Comparison of these results with the results of the MTT assays (Fig. 3) revealed that the cell lines that showed higher oxidative stress presented higher rates of apoptosis and lower IC_50_ values.

Kim *et al*.^42^, found that cells exposed to AgNPs presented regulation of mRNA expression related to oxidative stress that differed from the effect of exposure to Ag^+^ ions, suggesting that the toxicity induced by AgNPs could be an intrinsic effect, independent of the action of free Ag^+^ ions, with the mechanisms of action being different. Although many studies have reported the toxicity of silver nanoparticles towards different cell types, the characteristics of the nanoparticles may vary from study to study. Nonetheless, the AgNP-T studied here seemed to behave similarly, in terms of the general response characteristics, to other nanoparticles studied to date.

In general, the effect of oxidative stress on apoptosis is associated with DNA damage in the cell. The assessment of DNA damage was therefore performed using comet analyses.

### Comet assay

**Figure 5:**
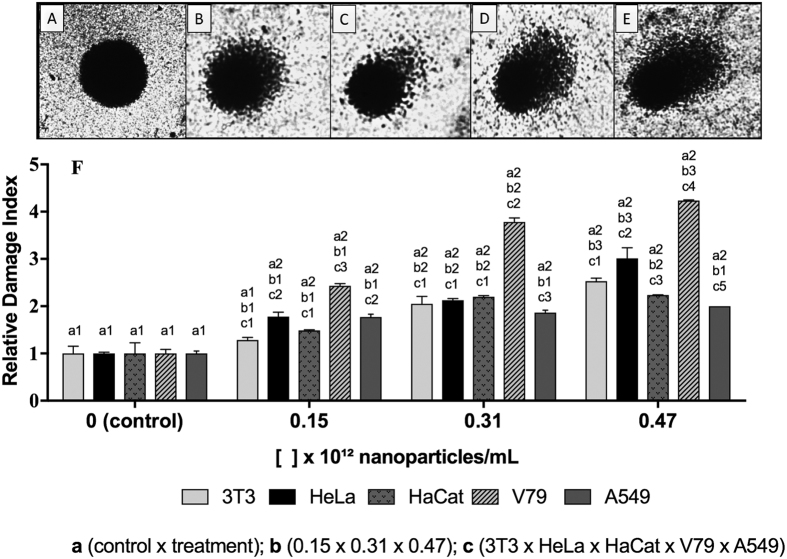
Analysis of DNA damage using the comet test applied to 3T3, HeLa, HaCaT, V79 and A549 cells exposed to the biogenic silver nanoparticles (AgNP-T) for 1 h at 37 °C. Scores: (**A**) 0; (**B**) 1; (**C**) 2; (**D**) 3; (**E**) 4 (100x magnification), where 0 indicates absence of damage and 4 indicates maximum damage; (**F**) Relative damage indices for AgNP-T at different concentrations and using different cell lines. Different letters indicate different comparisons and different numbers indicate significant differences (p < 0.05).

The comet assay was used to determine the genotoxicity of the biogenic silver nanoparticles. The results showed that the nanoparticles were genotoxic towards the five cell lines, which showed significantly higher damage indices, compared to the negative control (Fig. 5), although the responses varied according to the cell line.

The concentrations used in the comet assays were lower than employed in the oxidative stress and necrosis/apoptosis analyses, because the aim was to assess the genotoxicity of the nanoparticles at concentrations likely to be used in the field. The findings indicated that exposure to AgNP-T caused DNA damage that could be repaired, although considering the results of the oxidative stress and necrosis/apoptosis analyses, the results might be indicative of the non-recovery of some cell lines.

Panda *et al*.^26^, investigated the effects of biogenic silver nanoparticles using *Allium cepa* cells and comet assays, and observed genotoxicity when compared to the negative control. Lima *et al*.^27^, exposed human lymphocytes and 3T3 cells to biogenic silver nanoparticles synthesized from *Fusarium oxysporum* and observed no genotoxic effects at the concentrations tested, using comet assays. It is important to point out that due to different synthesis conditions and the use of various reducing agents, biogenic silver nanoparticles can present different characteristics in terms of parameters such as size and morphology, as well as cytotoxic and genotoxic potential^22^,^46^. In the present work, the results of the comet assays were consistent with the *Allium cepa* tests, in which significantly higher alteration index values were found for cells exposed to the biogenic silver nanoparticles, compared to the negative control.

### Minimum inhibitory concentration (MIC)

The results of the MIC assays showed that AgNP-T presented inhibitory potential against the pathogens *Escherichia coli, Staphylococcus aureus*, and *Candida albicans*, with minimum inhibitory concentrations of 0.31 × 10^12^, 0.31 × 10^12^, and 0.47 × 10^12^ NPs/mL, respectively. The ability of biogenic nanoparticles to control these species of pathogenic microorganisms has been observed previously in several studies, encouraging further investigation of this capacity^47^,^48^. In the present work, evaluation was also made of the inhibitory potential of AgNP-T towards a pool of bacteria extracted from soil. The results showed that this bacterial pool presented greater resistance, compared to the isolated pathogenic species, with MIC of 1.58 × 10^12^ NPs/mL. This greater resistance of the bacterial pool could have been because the different species of microorganisms developed associations that conferred greater resistance to the AgNPs, compared to isolated strains^49^. It is expected that soil bacteria would show similar resistance when exposed to AgNP-T in the environment.

### Effects of the nanoparticles on bacteria involved in the nitrogen cycle

Molecular analysis of the bacterial genes involved in the nitrogen cycle enabled assessment of the effects of AgNP-T on the quantity and distribution of the bacteria that participate in the steps of nitrogen fixation, nitrification, and denitrification. The nitrogen cycle bacteria are among the most important microorganisms for agriculture, making the soil fertile by converting nitrogen into bioavailable forms that can be assimilated by living beings for production of organic molecules such as proteins and amino acids^50^.

**Figure 6:**
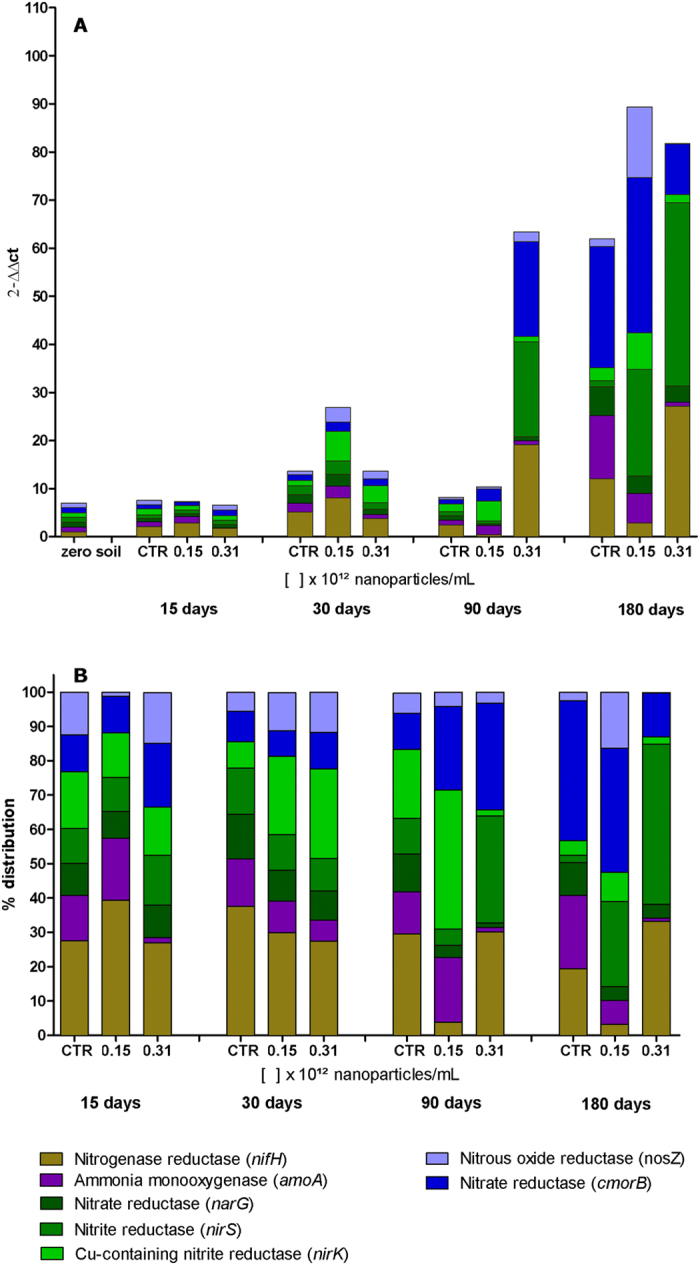
Quantitative molecular analysis of genes of bacteria involved in the nitrogen cycle (*nifH, amoA, nosZ, cmorB, nirK, narG*, and *nirS*) in soils exposed to the biogenic silver nanoparticles (AgNP-T) at different concentrations, after 15, 30, 90, and 180 days post-treatment. (**A**) Quantities of genes of bacteria associated with the nitrogen cycle; (**B**) Proportions of genes of bacteria associated with the nitrogen cycle. The zero soil was assigned a value of 1 and the other samples were quantified using this as a reference.

Mishra and Kumar^51^, reviewed several studies in which metal nanoparticles were exposed to the environment, concluding that they could cause toxicity towards soil bacteria that contribute to the development of plants. Little is known about the effects of silver nanoparticles on the nitrogen cycle bacteria. Here, quantitative molecular assessments of soils treated with AgNP-T were performed, considering the initial soil used in the experiments as a reference sample (denoted soil zero). The 16S rRNA gene was used as a genetic reference, as described by Watanabe *et al*.^52^. The soil microbiota are sensitive to physical factors such as temperature and humidity, so even in soils without exposure to the nanoparticles, there could be differences in terms of the numbers and types of bacteria studied (Fig. 6).

The quantity of bacteria increased over time in all the soil samples, with the samples exposed to AgNP-T exhibiting a greater increase, compared to the control sample. After 15 days, there were no significant changes in the numbers and distributions of the bacteria. After 30 days, the soil exposed to AgNP-T at a concentration of 0.15 × 10^12^ NPs/mL showed the greatest difference in terms of the number of bacteria, with no major difference in the distribution of the different types of bacteria, compared to the control (Fig. 6A and B). However, after 90 days, differences in the numbers of bacteria as well as their distributions became more evident, with decreases in the bacteria producing nitrogenase reductase enzymes (*nifH*) and nitrate reductases of the first phase of denitrification (*narG*). For exposure at 0.15 × 10^12^ NPs/mL, the decrease in the nitrogenase-reductases (*nifH*) was maintained until the end of the experiment (at 180 days). It could also be seen that for both exposure concentrations, there were changes in the distributions of the types of bacteria.

The bacteria that presented nitrate reductase (*narG*), an enzyme involved in the first stage of denitrification, decreased during the post-exposure period, while the bacteria that exhibited the other enzymes of the first phase (*nirS* and *nirK*) showed oscillations in their proportions. Bacteria that presented the *cmorB* nitrate reductase genes increased up to 90 days post-exposure and decreased after this period, while the bacteria that presented the nitrous oxide reductase gene (*nosZ*) oscillated in the opposite way, increasing for the first two periods and decreasing for the last two periods (90 and 180 days). A likely explanation for the lack of any changes during the initial days of exposure was that the biogenic nanoparticles possessed a coating, which could have delayed the onset of action of the nanoparticles and the release of the Ag^+^ ions that were probably responsible for the effects on the microbiota.

Studies involving the molecular analysis of soil microbiota and the effects of exposure to nanomaterials are still in the early stages, with further investigation needed in relation to environmental parameters. Recent studies have reported the need to develop new primers for these assessments, in order to ensure inclusion of all the bacteria presenting genes responsible for the synthesis of enzymes that participate in the nitrogen cycle^53^,^54^.

The results obtained in the present work were similar to the findings of Yang *et al*.^55^, who investigated the effects of silver nanoparticles on the nitrogen cycle bacteria *Pseudomonas stutzeri* (denitrifying), *Azotobacter vinelandii* (nitrogen fixing), and *Nitrosomonas europaea* (nitrifying), using the minimum inhibitory concentration (MIC) test and analysis of gene expression by RT-qPCR. The results indicated that the denitrifying and nitrogen fixing bacteria showed greater resistance to the silver nanoparticles, while *Nitrosomonas europaea* showed the greatest susceptibility, with MIC values lower than those of the bacteria of the other steps of the cycle, as well as changes in gene expression.

### Potential of the biogenic silver nanoparticles to control white mold and possible effects in *Trichoderma harzianum*

**Figure 7:**
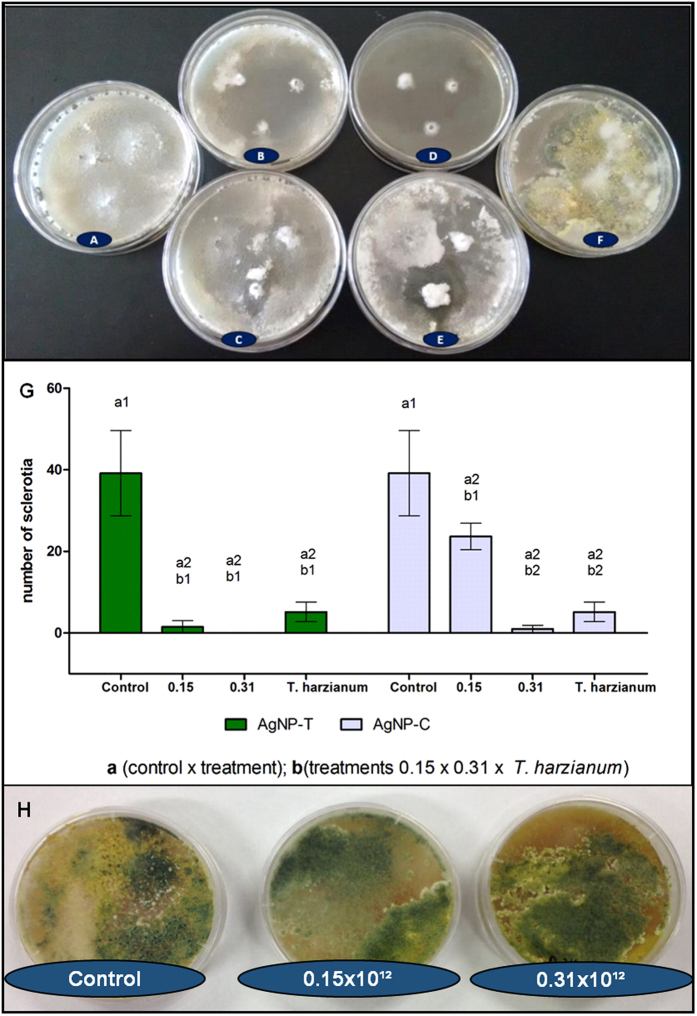
Appearance of the white mold cultures grown in Petri dishes on PDA medium supplemented with biogenic silver nanoparticles (AgNP-T) or commercial silver nanoparticles (AgNP-C) for 14 days at room temperature and with a photoperiod of 12 h. (**A**) control; (**B**) AgNP-T at 0.15 × 10^12^ NPs/mL; (**C**) AgNP-C at 0.15 × 10^12^ NPs/mL; (**D**) AgNP-T at 0.31 × 10^12^ NPs/mL, (**E**) AgNP-C at 0.31 × 10^12^ NPs/mL, (**F**) *T. harzianum* at 0.127 mg/mL; (**G**) Formation of new sclerotia in different treatments and control (different letters indicate different comparisons and different number indicate significant statistical differences p < 0.05); (**H**) Evaluation of *Trichoderma harzianum* culture exposed to AgNP-T at 0.15 and 0.31 × 10^12^ NPs/mL.

The potential of AgNP-T to control the white mold fungus was evaluated by plating the sclerotia on Potato-Dextrose Agar (PDA) and applying treatments of the biogenic silver nanoparticles at concentrations of 0.15 × 10^12^ and 0.31 × 10^12^ NPs/mL (Fig. 7B and D). A control culture consisted of the culture medium alone (Fig. 7A).

The results showed that at a concentration of 0.31 × 10^12^ NPs/mL, the biogenic nanoparticles inhibited mycelial growth and the formation of new sclerotia of *Sclerotinia sclerotiorum*, with the sclerotia placed on PDA supplemented with nanoparticles remaining surrounded by a small quantity of mycelium, without the formation of new sclerotia (Fig. 7D).

In the cultures supplemented with biogenic nanoparticles at a concentration of 0.15 × 10^12^ NPs/mL (Fig. 7B), mycelium was released from the precursor sclerotia and new sclerotia were formed. In the cultures treated with *Trichoderma harzianum* (Fig. 7F), there was release of mycelium and the formation of new sclerotia. There was colonization of *T. harzianum* on the sclerotia, which became surrounded by the biological control agent, although the colonization did not prevent the germination of sclerotia, so that the cultures became mixed. In the control cultures, where the sclerotia were placed on untreated PDA, mycelia grew over the entire plate, and several new sclerotia were formed (Fig. 7A). The means of the formation of new sclerotia in different treatments and control are shown in Fig. 7G.

For comparison, analyses were performed using the commercial silver nanoparticles (Fig. 7C and E), which produced inferior results, compared to use of the biogenic nanoparticles. The inhibitory potential of the biogenic silver nanoparticles at a concentration 0.31 × 10^12^ NPs/mL on the germination of sclerotia appeared to represent a possible alternative for the control of white mold. According to Le Tourneau^56^, control of this disease can be achieved by interrupting the life cycle of the phytopathogenic fungus, preventing the formation and germination of the resistance structures (the sclerotia).

Evaluating possible inhibitory effects of the biogenic silver nanoparticles on *Trichoderma harzianum* culture no deleterious effects were observed in both the concentrations tested in comparison with negative control (Fig. 7H). It means that if *T. harzianum* were applied combined with the nanoparticles this fungus development would not be affected.

### Effects of the silver nanoparticles on the germination and growth of soybean

**Figure 8:**
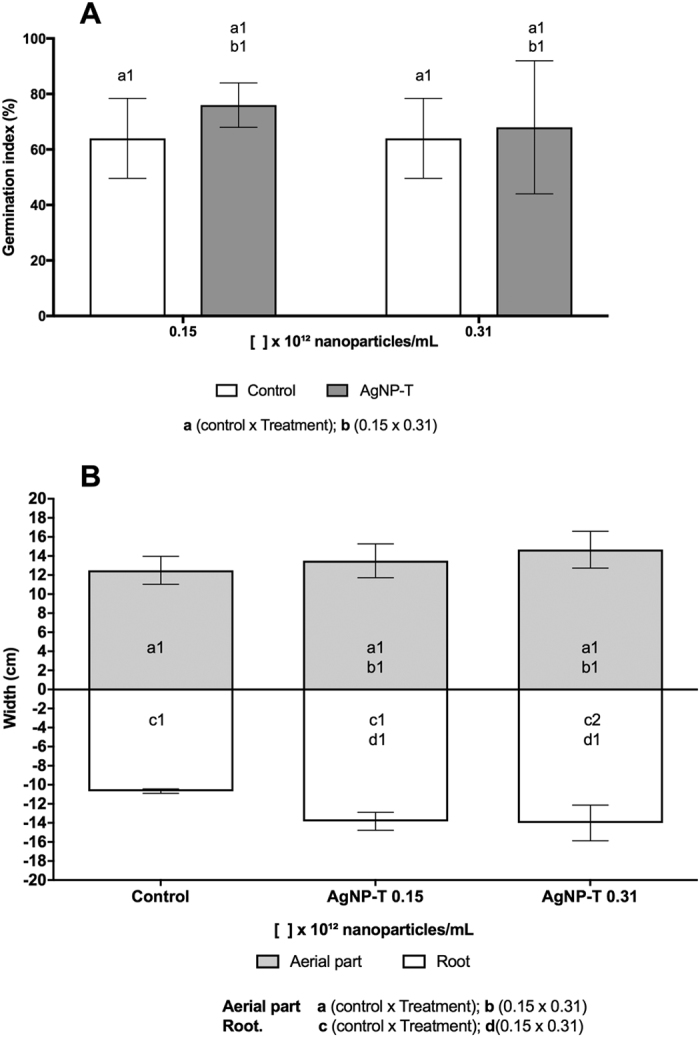
Effect of different concentrations of biogenic silver nanoparticles (AgNP-T) on soybean cultures. (**A**) Germination index (%) of seeds sown in Petri dishes and incubated at 25 °C in the dark for 7 days; (**B**) Growth of aerial parts and roots. Different letters indicate different comparisons and different number indicate significant statistical differences (p < 0.05).

Experiments were performed to determine the effects of the AgNP-T biogenic nanoparticles on the germination and seedling growth of soybean. Exposure of the beans to the biogenic silver nanoparticles, at all the concentrations tested, did not result in any significant differences compared to the negative control (Fig. 8A and B).

These findings suggest that the treatment of soybean seeds with the nanoparticles would be unlikely to induce any inhibitory effects on the development of the crop.

## Conclusions

Although nanotechnology is a recent introduction in the agricultural sector, there are several ways in which it can contribute to both increased productivity and pest control, while at the same time helping to protect the environment. The present work demonstrated that it is possible to perform the biogenic synthesis of silver nanoparticles using *T. harzianum* as the reducing agent, and that the nanoparticles exerted inhibitory action on the germination of the resistance structures of the *Sclerotinia sclerotiorum* pathogen. In terms of toxicity, comparison with the controls showed that the biogenic nanoparticles presented cytotoxic and genotoxic effects that varied according to the cell line used and the exposure concentration. An important finding was that in most of these assays, the effects increased in direct proportion to the concentrations used, and were most intense at concentrations above those used in the assessments of inhibition of *S. sclerotiorum*. Concerning the effects of the nanoparticles in the soil, there was evidence of some partial effects on the bacteria, with possible recovery of the microbiota over time. The AgNP-T nanoparticles did not present any negative effects on the germination and growth of soybeans, from which it could be concluded that this technique represents a first step towards as potential control of white mold in soybean crops using nanotechnology.

## Methods

### Culture of *Trichoderma harzianum* and biogenic synthesis of silver nanoparticles

The culture of *Trichoderma harzianum* employed the product Ecotrich^TM^, used for biological control of phytopathogens. The concentration used in the field (0.127 mg/mL) was plated onto PDA medium, followed by culturing at room temperature, in the dark, for 6 days. After growth, mycelium discs were transferred to Potato-Dextrose Broth (PD) and cultured for 12 days, with stirring at 150 rpm^57^. The biomass was filtered, transferred to ultrapure water, and maintained under agitation for 72 h, then vacuum filtered and discarded. Silver nitrate (1 × 10^−3^ mol L^−1^) was added to the filtrate which was kept under agitation until color change from light yellow to dark brown, confirming the synthesis^58^.

### Physico-chemical characterization

Physico-chemical characterization was performed by dynamic light scattering (DLS), zeta potential using microelectrophoresis, nanoparticle tracking analysis (NTA), and scanning electron microscopy (SEM) coupled with energy dispersive spectroscopy (EDS). The DLS and microelectrophoresis analyses employed a Zetasizer Nano 90 instrument (Malvern Instruments, UK). Readings were performed in triplicate at ambient temperature (25 °C). The NTA analyses employed a NanoSight LM14 instrument with NanoSight v. 2.3 software, and the samples were diluted 10,000 times. For SEM and EDS analyses, thin films were prepared by dripping undiluted nanoparticles onto silicon grids, which were kept in a desiccator for 24 h. The samples were then analyzed by SEM (JSM-6701F, JEOL) at 5 kV energy and with spot size between 3.0 and 4.0.

### *Allium cepa* chromosome aberration assays

Roots of *Allium cepa* were placed in contact for 24 h with the nanoparticles at concentrations of 3.16, 1.58, 0.31, and 0.15 × 10^12^ NPs/mL, and in distilled water as negative control. The roots were then fixed in ethanol:acetic acid (3:1) for 24 h, followed by hydrolysis with 1 M HCl for 9 min in a water bath at 60 °C. The roots were stained with Schiff reagent for 2 h, followed by slides preparation with 2% acetic carmine staining. The analyses were performed with an optical microscope at 40x magnification, considering the total number of cells, the number of cells in division, and the number of alterations. Calculations were then made of mitotic index (MI, the number of cells in division/total number of cells counted) and alteration index (AI, the number of cells with chromosomal alterations/number of cells in division). The same indices were also calculated relative to the negative control, giving the relative indices (MIr and AIr). The tests were performed in triplicate with three repetitions.

### Cell viability evaluation using the reduction of methylthiazol tetrazolium (MTT)

Cells from lines 3T3 (mouse embryo fibroblasts), HeLa (human cervical adenocarcinoma), HaCaT (human keratinocytes), V79 (Chinese hamster pulmonary fibroblasts), and A549 (human epithelial adenocarcinoma) were placed in 96-well plates (2 × 10^4^ cells/well), incubated at 37 °C until adherence, and exposed to decreasing concentrations of the nanoparticles for 24 h. The cultures were then washed with PBS, followed by addition to each well of 100 μL of MTT solution (3-(4,5-dimethylthiazolyl-2)-2,5-diphenyltetrazolium bromide) (5 mg/mL). The cells were incubated for 3 h (5% CO_2_, 37 °C), followed by fixing in 100 μL of ethanol. Two controls were used, one with untreated cells and one with MTT only (without cells). The cell viability was determined using an ELISA reader (Thermo fisher EVOLUTION 201) at 570 nm. The assays with which one of the cell lines were performed in sextuplicates with three repetitions.

### Imaging cytometry – Tali^TM^

Analyses of cell viability, necrosis, and apoptosis were performed using the Tali™ Apoptosis Kit - Annexin V AlexaFluor^®^ 488 and Propidium Iodide (Invitrogen). The cells were previously treated with the biogenic nanoparticles at a concentration of 0.93 × 10^12^ NPs/mL for 24 h, and samples were prepared according to the kit specifications. The reading was performed with the Tali™ Image-Based Cytometer.

The oxidative stress analyses were performed using CellRox Orange^TM^ reagent (Invitrogen). The cells were previously treated with the biogenic nanoparticles at a concentration of 0.93 × 10^12^ NPs/mL for 1 h, and samples were prepared according to the kit specifications. The reading was performed with the Tali™ Image-Based Cytometer. Imaging cytometry assays with each one of the cell lines were performed thrice.

### Comet assay

The comet assays were performed using an adaptation of the methodology described by Singh *et al*.^59^. Cells from the lines 3T3, HeLa, HaCaT, V79 and A549 were maintained in contact with the nanoparticles at concentrations of 0.15 × 10^12^, 0.31 × 10^12^, and 0.47 × 10^12^ NPs/mL for 1 h. Cells incubated without any treatment were used as negative control. After exposure, cells were homogenized with 0.8% low melting point agarose, applied to slides coated with standard 1.5% agarose, covered with cover slips, and kept in refrigerator until solidified. The slides were prepared in duplicate. Subsequently, the cover slips were removed, the slides were kept in ice-cold lysis solution for 1 h, neutralized for 5 min, covered with an alkaline buffer for 20 min, and submitted to electrophoresis at 4 °C for 20 min at 300 mA (1.6 V/cm). After electrophoresis, the slides were neutralized for 5 min, dried at ambient temperature and fixed for 10 min. For staining, the slides were hydrated and placed in staining solution for 35 min. Cells were analyzed using an optical microscope, by visual scoring, classifying the damage in categories from 0 to 4, where 0 represents an absence of damage and 4 corresponds to the highest level of damage. Approximately 100 randomly selected cells were analyzed per slide, totaling 200 cells per treatment. The assays with each one of the cell lines were performed with three repetitions.

### Minimum inhibitory concentration (MIC)

The microorganisms *Escherichia coli, Staphylococcus aureus*, and *Candida albicans*, and a pool of bacteria extracted from soil, were allowed to grow in Mueller Hinton culture broth for 24 h. Counting was then performed in a Neubauer chamber and the cells were diluted to a concentration of 5 × 10^5^ CFU/mL. The assay was performed in 96-well plates. To each well were added 10 μL of medium containing the microorganisms, together with the nanoparticles at decreasing concentrations. The wells were completed with culture broth to a final volume of 90 μL, and the plate was incubated at 37 °C for 24 h. After this time, 10 μL of Resazurin solution (6.75 mg/mL) was added to each well and the plates were incubated at 37 °C for 24 h, prior to visual analysis of the color. The assays with which one of the microorganisms were performed in sextuplicates with three repetitions.

### Molecular analysis of nanoparticles effects on nitrogen cycle bacteria by qPCR

The soil used in this work was obtained from local agricultural supplier (14% organic composition, pH 6,8). Before use, the soil was sieved, separated into vessels, wetted and incubated for 30 days at 25 °C. After this period, the soil was exposed to the nanoparticles at concentrations of 0.15 × 10^12^ and 0.31 × 10^12^ NPs/mL. A separate untreated soil sample was used as the negative control. The samples were prepared in triplicate.

The extraction of DNA from soil microorganisms was performed using the Power Soil^®^ DNA Isolation Kit (MoBio Laboratories). On the day of exposure, DNA was extracted from a soil sample without any type of treatment (denoted soil zero) for use as a control reflecting the initial soil conditions. DNA extractions from the soil samples treated with nanoparticles, as well as the negative control, were performed 15, 30, 90, and 180 days after treatment. Quantification of the genetic material was performed by fluorescence (Qubit 3.0 fluorometer) using a Qubit dsDNA BR Assay Kit (Invitrogen). Using the concentrations obtained, the samples were diluted to final concentrations of 1000 ng/mL.

Quantification was made of specific genes from nitrogen cycle bacteria: *nifH* (nitrogen fixation), *amoA* (nitrification), *nirK, nirS*, and *narG* (first stage of denitrification), and *cmorB* and *nosZ* (second stage of denitrification). Quantitative evaluation employed real-time polymerase chain reactions (qPCR), with the samples and genes measured in triplicate using a StepOne thermocycler. The primers used and conditions for amplification were based on Jung *et al*.^60^. The reactions were performed in a final volume of 25 μL, containing 12.5 μL of Planium^TM^ SYBR^TM^ Green qPCR SuperMix-UDG with ROX (Invitrogen), 1 μL of sense primer, 1 μL of anti-sense primer, 1 μL of DNA sample, and sufficient ultrapure water to complete. Relative quantification of the DNA used the 16S rRNA gene as a reference, and the control was DNA extracted from soil zero. Soil zero was assigned a value of 1, and the other samples were quantified using this as a reference. The calculation employed was quantification according to ΔΔCt, constructing a calibration curve for each gene analyzed. The slopes obtained were between −2.9 and −3.4, with r^2^ close to 1, which ensured quality and confidence in the experiment.

### Effects of the silver nanoparticles on mycelium and sclerotia of *S. sclerotiorum* and on *Trichoderma harzianum* development

The initial inoculum of *S. sclerotiorum* was obtained from cabbage leaves containing mycelium and sclerotia. Sclerotia were placed in PDA Petri plates and kept for 7 days at ambient temperature, with a photoperiod of 12 h. After this period, the formation of white mycelium was observed, together with new sclerotia.

For evaluation of the potential of nanoparticles to control *S. Sclerotiorum*, Petri plates were prepared in duplicate with PDA supplemented with the nanoparticles at concentrations of 0.15 × 10^12^ and 0.31 × 10^12^ NPs/mL and *T. harzianum* (0.127 mg/mL), as well as control cultures containing PDA alone. Three sclerotia were arranged on each plate and cultures were performed for 14 days at ambient temperature, with a 12 h photoperiod. At the end of the culture, counting of new sclerotia was performed and presence or absence of mycelium was visually examined.

As *T. harzianum* is already used for white mold control the effects of the biogenic silver nanoparticles on this biocontrol fungus were also evaluated through its culture in PDA supplemented with the nanoparticles in the same concentrations above. Both the assays were performed in triplicate with three repetitions.

### Effects of the silver nanoparticles on soybean germination and growth

Soybean seeds were acquired in an organic food store. Decontamination was performed in 1% sodium hypochlorite for 2 min. The seeds were exposed to nanoparticles at concentrations of 0.15 × 10^12^ and 0.31 × 10^12^ NPs/mL, and ultrapure water was used as a negative control. For germination test, the seeds were placed on filter papers in Petri plates and incubated in the dark at 25 °C for 7 days. Five plates were prepared for each treatment, with five seeds per plate. The germination index was obtained by counting the germinated seeds. For the growth test, the seeds were planted in plastic tubes containing substrate. Two seeds were planted in each tube, with a total of 5 tubes per treatment. The seedlings were kept in a greenhouse for 15 days, with watering once a day. After growth period, the seedlings were removed from the substrate for measurements of aerial parts and roots. The germination and growth tests were performed thrice.

### Statistical Analysis

In toxicological assays, sclerotia development and soybean germination and growth, treatments were always compared with negative control employing analysis of variance (ANOVA) followed by Tukey’s *post hoc* test using GraphPad Prism v. 7.0 software. The statistical significance was p < 0.05.

In molecular analysis of nitrogen cycling bacteria the StepOne thermocycler software already supplies data with statistical analysis. The molecular quantification was realized using method ΔΔCT (2^−ΔΔCT^), including sample (zero soil) as control sample and 16sr RNA as control gene. The statistic equations are showed above (Equation 1 and 2)


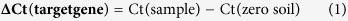



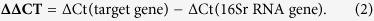


## Additional Information

**How to cite this article**: Guilger, M. *et al*. Biogenic silver nanoparticles based on *trichoderma harzianum*: synthesis, characterization, toxicity evaluation and biological activity. *Sci. Rep.* **7**, 44421; doi: 10.1038/srep44421 (2017).

**Publisher's note:** Springer Nature remains neutral with regard to jurisdictional claims in published maps and institutional affiliations.

## References

[b1] Bruinsma J. The resource outlook to 2050: by how much do land, water and crop yields need to increase by 2050. Expert Meeting on How to Feed the World in 2050, FAO, 1–33 (2009).

[b2] FIESP. Federação das Indústrias do Estado de São Paulo. Safra mundial de soja 2016/2017. *Informativo Deagro*. (Mai, 2016).

[b3] Masuda T. & Goldsmith P. D. World soybean production: area harvested, yield, and long-term projections. Int. Food Agribus. Manag. Rev. 12, 143–161 (2009).

[b4] Bolton M. D., Thomma B. P. H. J. & Nelson B. D. *Sclerotinia sclerotiorum* (lib.) de bary: biology and molecular traits of a cosmopolitan pathogen. Mol. Plant Pathol. 7, 1–16 (2006). 20507424 10.1111/j.1364-3703.2005.00316.x

[b5] Esker P. . Management of white mold in soybean. North Central Soybean Research Program (2011).

[b6] Watson Q. & Smith D. Disease profile: sclerotinia stem rot of soybean. University of Wisconsin – Extension (2013).

[b7] Fraceto L. F. . Nanotechnology in agriculture: which innovation potential does it have? Front. Environ. Sci. 4, 1–5 (2016).

[b8] Grillo R. . Poly(epsilon-caprolactone)nanocapsules as carrier systems for herbicides: physico-chemical characterization and genotoxicity evaluation. J. Hazard. Mater. 231, 1–9 (2012). 22795586 10.1016/j.jhazmat.2012.06.019

[b9] Grillo R. . Chitosan/tripolyphosphate nanoparticles loaded with paraquat herbicide: an environmentally safer alternative for weed control. J. Hazard. Mater. 278, 163–171 (2014). 24968252 10.1016/j.jhazmat.2014.05.079

[b10] Grillo R. . Chitosan nanoparticles loaded the herbicide paraquat: the influence of the aquatic humic substances on the colloidal stability and toxicity. J. Hazard. Mater. 286, 562–572 (2015). 25636059 10.1016/j.jhazmat.2014.12.021

[b11] Grillo R., Abhilash P. C. & Fraceto L. F. Nanotechnology applied to bio-encapsulation of pesticides. J. Nanosci. Nanotechnol. 16, 1231–1234 (2016). 27398594 10.1166/jnn.2016.12332

[b12] Maruyama C. R. . Nanoparticles based on chitosan for the combined herbicides imazapic and imazapyr. Sci. Rep. 6, 1–13 (2016). 26813942 10.1038/srep19768 PMC4728438

[b13] Vamvakaki V. & Chaniotakis N. A. Pesticide detection with a liposome-based nanobiosensor. Biosens. Bioelectron. 22, 2848–2853 (2007). 17223333 10.1016/j.bios.2006.11.024

[b14] Kim S. W. . Antifungal effects of silver nanoparticles (agnps) against various plant pathogenic fungi. Microbiology 40, 53–58 (2012). 10.5941/MYCO.2012.40.1.053 PMC3385153 22783135

[b15] Rao K. J. & Paria S. Use of sulfur nanoparticles as a green pesticide on *fusarium solani* and *venturia inaequalis* phytopathogens. RSC Adv. 3, 10471–10478 (2006).

[b16] Mishra S. . Biofabricated silver nanoparticles act as a strong fungicide against *bipolaris sorokiniana* causing spot blotch disease in wheat. PloS One 9, 1–11 (2014). 10.1371/journal.pone.0097881 PMC4026416 24840186

[b17] Marin S. . Applications and toxicity of silver nanoparticles: a recent review. Curr. Topic. Med. Chem. 15, 1596–1604 (2015). 10.2174/1568026615666150414142209 25877089

[b18] Tran Q. U., Nguyen V. Q. & Le A. T. Silver nanoparticles: synthesis, properties, toxicology, applications and perspectives. Adv. Nat. Sci. Nanosci. Nanotechnol. 4, 1–21 (2013).

[b19] Prabhu S. & Poulose E. K. Silver nanoparticles: mechanism of antimicrobial action, synthesis, medical applications, and toxicity effects. Int. Nano Lett. 2, 1–10 (2012).

[b20] Dhillon G. S., Brar S. K., Kaur S. & Verma M. Green approach for nanoparticle biosynthesis by fungi:current trends and applications. Crit. Rev. Biotechnol. 32, 49–73 (2011). 21696293 10.3109/07388551.2010.550568

[b21] Singh P. & Raja R. B. Biological synthesis and characterization of silver nanoparticles using the fungus trichoderma harzianum. Asian J. Exp. Biol. Sci. 2, 600–605 (2011).

[b22] Ahluwalia V., Kumar J., Sisodia R., Shakil N. A. & Walia S. Green synthesis of silver nanoparticles by *trichoderma harzianum* and their bio-efficacy evaluation against *staphylococcus aureus* and *klebsiella pneumonia*. Ind. Crops Prod. 55, 202–206 (2014).

[b23] Nanocomposix. Guidelines for dynamic light scattering measurement and analysis. Nanocomposix 1.3, 1–7 (2012).

[b24] Gade A. . Exploitation of *aspergillus niger* for synthesis of silver nanoparticles. J. Biobased Mater. Bioenergy 2, 243–247 (2008).

[b25] Iravani S., Korbekandi H., Mirmohammadi S. V. & Zolfaghari B. Synthesis of silver nanoparticles: chemical, physical and biological methods. Res. Pharm. Sci. 9, 385–406 (2014). 26339255 PMC4326978

[b26] Panda K. K. . *In vitro* biosynthesis and genotoxicity bioassay of silver nanoparticles using plants. Toxicol. In Vitro 25, 1097–1105 (2011). 21419840 10.1016/j.tiv.2011.03.008

[b27] Lima R. . Cytotoxicity and genotoxicity of biogenic silver nanoparticles. J. Phys.: Conf. Ser. 1–9 (2013).

[b28] Braroo K. . Colloidal silver nanoparticles from *ocimum sanctum*: synthesis, separation and their implications on pathogenic microorganisms, human keratinocyte cells, and *allium cepa* root tips. J. Colloid Sci. Biotechnol. 3, 245–252 (2014).

[b29] Kumari M., Mukherjee A. & Chandrasekaran N. Genotoxicity of silver nanoparticles in allium cepa. Sci. Total Environ. 407, 5243–5246 (2009). 19616276 10.1016/j.scitotenv.2009.06.024

[b30] Badu K., Deepa M., Shankar S. & Rai S. Effect of nano-silver on cell division and mitotic chromosomes: a prefatory siren. Internet J. Nanotechnol. 2, 1–7 (2007).

[b31] Lima R., Seabra A. B. & Durán N. Silver nanoparticles: a brief rewiew of cytotoxicity and genotoxicity of chemically and biogenically synthesized nanoparticles. J. Appl. Toxicol. 32, 867–879 (2012). 22696476 10.1002/jat.2780

[b32] Mukherjee S. G., O’claonadh N., Casey A. & Chambers G. Comparative *in vitro* cytotoxicity study of silver nanoparticle on two mammalian cell lines. Toxicol. In Vitro 26, 238–251 (2012). 22198051 10.1016/j.tiv.2011.12.004

[b33] Han J. W. . Oxidative stress mediated cytotoxicity of biologically synthesized silver nanoparticles in human lung epithelial adenocarcinoma cell line. Nanoscale Res. Lett. 9, 1–14 (2014). 25242904 10.1186/1556-276X-9-459 PMC4167841

[b34] Monteiro-Riviere N. A., Inman A. O. & Zhang L. W. Limitations and relative utility of screening assays to assess engineered nanoparticle toxicity in a human cell line. Toxicol. Appl. Pharmacol. 234, 222–235 (2009). 18983864 10.1016/j.taap.2008.09.030

[b35] Prina-Mello A. . Comparative flow cytometric analysis of immunofunctionalized nanowire and nanoparticle signatures. Small 6, 247–255 (2010). 19941303 10.1002/smll.200901014

[b36] Greulich C. . Uptake and intracellular distribution of silver nanoparticles in human mesenchymal stem cells. Acta Biomater. 7, 347–354 (2011). 20709196 10.1016/j.actbio.2010.08.003

[b37] Kumar G., Degheidy H., Casey B. J. & Goering P. L. Flow cytometry evaluation of *in vitro* cellular necrosis and apoptosis induced by silver nanoparticles. Food Chem. Toxicol. 85, 45–51 (2015). 26115599 10.1016/j.fct.2015.06.012

[b38] Mizushima N. Autophagy: process and function. Genes Dev. 21, 2861–2873 (2007). 18006683 10.1101/gad.1599207

[b39] Zhang X. F. . Differential nanoreprotoxicity of silver nanoparticles in male somatic cells and spermatogonial stem cells. Int. J. Nanomed. 10, 1335–1357 (2015). 10.2147/IJN.S76062 PMC4337509 25733828

[b40] Kang S. J., Lee Y. J., Lee E. K. & Kwak M. K. Silver nanoparticles-mediated g2/m cycle arrest of renal epithelial cells is associated with nrf2-gsh signaling. Toxicol. Lett. 211, 334–341 (2012). 22546375 10.1016/j.toxlet.2012.04.016

[b41] Franco-Molina M. A. . Antitumor activity of colloidal silver on mcf-7 human breast cancer cells. J. Exp. Clin. Cancer Res. 29, 1–7 (2010). 21080962 10.1186/1756-9966-29-148 PMC2996348

[b42] Kim S. . Oxidative stress-dependent toxicity of silver nanoparticles in human hepatoma cells. Toxicol. In Vitro, 23, 1076–1084 (2009). 19508889 10.1016/j.tiv.2009.06.001

[b43] Arora S., Jain J., Rajwade J. M. & Paknikar K. M. Interactions of silver nanoparticles with primary mouse fibroblasts and liver cells. Toxicol. Appl. Pharmacol. 236, 310–318 (2009). 19269301 10.1016/j.taap.2009.02.020

[b44] Carlson C. . Unique cellular interaction of silver nanoparticles: size-dependent generation of reactive oxygen species. J. Phys. Chem. 112, 13608–13619 (2008). 10.1021/jp712087m 18831567

[b45] Sriram N., Kalayarasan S. & Sudhandiran G. Enhancement of antioxidant defense system by epigallocatechin-3-gallate during bleomycin induced experimental pulmonary fibrosis. Biol. Pharm. Bull. 31, 1306–1311 (2008). 18591765 10.1248/bpb.31.1306

[b46] Kaviya S., Santhanalakshmi J. & Viswanathan B. Green synthesis of silver nanoparticles using polyalthia longifolia leaf extract along with d-sorbitol: study of antibacterial activity. J. Nanotechnol. 1–5 (2011).

[b47] Li G. . Fungus-mediated green synthesis of silver nanoparticles using *aspergillus terreus*. Int. J. Mol. Sci. 13, 466–476 (2012). 22312264 10.3390/ijms13010466 PMC3269698

[b48] Amaladhas T. P., Sivagami S., Devi T. A., Ananthi N. & Velammal S. P. Biogenic synthesis of silver nanoparticles by leaf extract of *cassia augustifolia*. Adv. Nat. Sci.: Nanosci. Nanotechnol. 3, 1–8 (2012).

[b49] Lee H. H., Molla M. N., Cantor C. R. & Collins J. J. Bacterial charity work leads to population-wide resistance. Nature Letters 467, 82–86 (2010). 10.1038/nature09354 PMC2936489 20811456

[b50] Hirsch P. R. & Mauchline T. H. The importance of the microbial n cycle in soil for crop plant nutrition. Adv. Appl. Microbiol. 93, 45–71 (2015). 26505688 10.1016/bs.aambs.2015.09.001

[b51] Mishra V. K. & Kumar A. Impact of metal nanoparticles on the plant growth promoting rhizobacteria. Dig. J. Nanomater. Bios. 4, 587–592 (2009).

[b52] Watanabe K., Kodama Y. & Harayama S. Design and evaluation of pcr primers to amplify bacterial 16s ribosomal dna fragments used for community fingerprinting. J. Microbiol. Methods 44, 253–262 (2001). 11240048 10.1016/s0167-7012(01)00220-2

[b53] Rusch A. Molecular tools for the detection of nitrogen cycling archaea. Archaea 2013, 1–10 (2013). 10.1155/2013/676450 PMC3556428 23365509

[b54] Wei W. . Higher diversity and abundance of denitrifying microorganisms in environments than considered previously. ISME J. 9, 1954–1965 (2015). 25756678 10.1038/ismej.2015.9 PMC4542046

[b55] Yang Y., Wang J., Xiu Z. & Alvarez P. J. J. Impacts of silver nanoparticles on cellular and transcriptional activity of nitrogen-cycling bacteria. Environ. Toxicol. Chem. 32, 1488–1494 (2013). 23554086 10.1002/etc.2230

[b56] Le Tourneau D. Morphology, citology, and physiology of sclerotinia species in culture. Phytopathology 69, 887–890 (1979).

[b57] Ávila Z. R. . Seleção de isolados de trichoderma spp. antagônicos a *sclerotinia rolfsii* e *sclerotinia sclerotiorum*. Embrapa Recursos Genéticos e Biotecnologia 117–130 (2005).

[b58] Durán N., Marcato P. D., De Souza G. I. H., Alves O. L. & Esposito E. Antibacterial effect of silver nanoparticles produced by fungal process on textile fabrics and their effluent treatment. J. Biomed. Nanotechnol. 3, 203–208 (2007).

[b59] Singh N. P., Mccoy M. T., Tice R. R. & Schneider E. L. A simple technique for quantification of low levels of dna damage in individual cells. Exp. Cell Res. 175, 184–191 (1988). 3345800 10.1016/0014-4827(88)90265-0

[b60] Jung J. . Change in gene abundance in the nitrogen biogeochemical cycle with temperature and nitrogen addition in antarctic soils. Res. Microbiol. 162, 1018–1026 (2011). 21839168 10.1016/j.resmic.2011.07.007

